# Successful mobilization using a combination of plerixafor and G-CSF in pediatric patients who failed previous chemomobilization with G-CSF alone and possible complications of the treatment

**DOI:** 10.1186/1756-8722-5-14

**Published:** 2012-03-30

**Authors:** Kyung Taek Hong, Hyoung Jin Kang, Nam Hee Kim, Min Sun Kim, Ji Won Lee, Hyery Kim, Kyung Duk Park, Hee Young Shin, Hyo Seop Ahn

**Affiliations:** 1 Department of Pediatrics, Cancer Research Institute, Seoul National University College of Medicine, Seoul, Republic of Korea

**Keywords:** Plerixafor, Hematopoietic Stem Cell Mobilization, Pediatrics, Complications, Interstitial Lung Diseases

## Abstract

Peripheral blood stem cell (PBSC) mobilization, which uses plerixafor (AMD 3100), a newly developed specific inhibitor of the CXCR4 receptor, in combination with granulocyte-colony stimulating factor(G-CSF), has been shown to enhance the stem cell mobilization in adult patients, but pediatric data are scarce. We documented our experience with this drug in 6 Korean pediatric patients who had failed in chemomobilization, using G-CSF, alone. All patients were mobilized CD34^+ ^cells (median, 11.08 × 10^6^/kg: range, 6.34-28.97 × 10^6^/kg) successfully within 2 to 3 cycles of apheresis, without complications. A total of 7 autologous transplantations were performed, including 1 tandem transplantation. However, 2 patients with brain tumors showed severe pulmonary complications, including spontaneous pneumomediastinum. This is the first study of PBSC mobilization with plerixafor in Asian pediatric patients. Furthermore our study suggests that mobilization with plerixafor may be effective in Korean pediatric patients, who have previously been heavily treated and have failed PBSC mobilization with classical chemomobilization, using G-CSF. However, further studies are needed to examine the possible complications of autologous transplantation, using a mobilized plerixafor product in children.

## Letter to the editor

Plerixafor has been introduced for the mobilization of hematopoietic stem cells to peripheral blood, by interfering with the SDF1-CXCR4 interaction. Although it has been FDA-approved in adult patients with non-Hodgkin lymphoma or multiple myeloma [1,2], the pediatric data usage are scarce, particularly in Asian children [3-8].

We retrospectively reviewed all 6 patients (3 males, 3 females), who received plerixafor-based mobilization at our center after obtaining the Institutional Review Board approval (H-1108-103-374). They had all previously failed peripheral blood stem cell (PBSC) mobilization by chemotherapy and G-CSF. The patient's characteristics, previous treatments and mobilization chemotherapies are shown. (Table 1) All patients received G-CSF (10 μg/kg) for 4 days, without prior chemotherapy. Then plerixafor (240 μg/kg; Mozobil, Genzyme Inc, Naarden, The Netherlands) and G-CSF (10 μg/kg) were administered subcutaneously, at 10 and 2 hours before each apheresis. CD34^+ ^cells (median, 11.08 × 10^6^/kg; range, 6.34-28.97 × 10^6^/kg) were mobilized successfully in all patients, after 2 to 3 apheresis without immediate complications (for each apheresis: mean, 6.28 × 10^6^/kg: range, 3.17-14.49 × 10^6^/kg). Seven autologous stem cell transplantations were performed, including 1 tandem transplantation, and the results of engraftment were acceptable. (Table 2)

**Table 1:** Patient Demographics and Prior Treatment Received

Pt #	Dx	Sex	Age	Earlier chemotherapy	Earlier radiotherapy	1st line mobilization chemotherapy
1	NBL	M	11	CDDP + VP16 + ADR + CPM, CPM + Topo + VP16	No	CPM + VP16+ GCSF
2	MBL	F	11	CDDP + CPM + VCR, CPM + VCR, Carbo + VP16 + IFM + VCR	IFRT + CSI	Carbo + VP16 + IFM + VCR + GCSF
3	OSA	M	6	CDDP + ADR + MTX, IFM + ADR + MTX	No	CPM + VP16+ GCSF
4	OSA	F	10	CDDP + ADR + MTX, IFM + ADR + MTX, Gemcitabine + Doxetaxel, CPM + Topo + VP16, IFM + Carbo + VP16	No	CPM + VP16+ GCSF
5	MBL	M	10	CDDP + CPM + VCR, CPM + VCR	IFRT + CSI	Carbo + VP16 + IFM + VCR + GCSF
6	ES	F	15	VCR + ADR + CPM, IFM + VP16	No	CPM + VP16+ GCSF

*ADR *Doxorubicin, *Carbo *Carboplatin, *CDDP *Cisplatin, *CPM *cyclophosphamide, *CSI *Craniospinal axis irradiation, *ES *Ewing sarcoma, *G-CSF *Granulocyte colony stimulating factor, *IFM *Ifosphamide, *IFRT *Involved field radiation therapy, *LP *Leukapheresis, *MBL *Medulloblastoma, *MTX *Methotrexate, *NBL *Neuroblastoma, *OSA *Osteosarcoma, *Pt *Patient, *Topo *Topotecan, *VCR *Vincristine, *VP16 *Etoposide

**Table 2:** PBSC Collection and Engraftment

			Plerixafor-based PBSC collection				Engraftment		
Pt #	Prior CD34 yield (10^6^ CD34 + cells/kg)	Days for LP	CD34+ cells(10^6^/kg)	TNC (10^8^/kg)	Days for LP	TPL #	PBSC infused (10^6 ^CD34+ cells/kg)	ANC > 500/mL (days)	PLT > 20,000/mL (transfusion-independent)(days)
1	1.24	4	12.64	16.13	3	#1	5.91	10	35
						#2	3.48	9	197
2	0.18	2	9.52	11.2	2		4.8	10	26
3	1.68	2	6.81	10.67	2		8.49	10	15
4	0.7	3	6.34	15.1	2		7.04	N/A	N/A
5	4	4	28.97	20.25	2		17.78	10	N/A
6	0.8	3	15.25	17.83	2		3.74	12	18

*ANC *Absolute Neutrophil Count, *LP *Leukapheresis, *N/A *Not Applicable (due to early death), *PBSC *Peripheral Blood Stem Cell, *PLT *Platelet, *Pt*, Patient, *TNC *Total Nuclear Cells, *TPL *Transplantation

Patients #1, #3 and #6 were disease-free at the last follow-up (28, 12 and 3 months after transplantation, respectively), however, patient #4 died on day 3, due to sudden cardiac arrest. Interestingly, two medulloblastoma patients (patients #2 and #5) showed serious lung problems, which include spontaneous pneumomediastinum on day 56 and 11, died on day 102 and 89, respectively. The cause of death of both patients showed to be respiratory failure, of which, the pathogen was not revealed by bronchoalveolar lavage or lung biopsy. The pathologic finding was consistent with a diffuse alveolar damage. In our center, 6 other patients who underwent the same radiotherapy and autologous PBSC transplantation, with the same conditioning regimen, but did not receive plerixafor for mobilization, have not shown such fatal pulmonary complication (data not shown). Due to the plerixafor mobilization of the different cell populations, in comparison with G-CSF alone [9,10], unexpected complications might occur after infusion in susceptible recipients, like the 2 patients that were mentioned above who underwent irradiation around the thoracic area.

In conclusion, we report the first data of Asian pediatric usage of plerixafor for PBSC mobilization, which showed a success rate of 100%, without acute complications. This is a higher success rate than those in the previous pediatric studies [3,7]. However we experienced two patients with medulloblastoma, who suffered from fatal pulmonary complication. Though the pathogenesis was not understood, further studies are needed to investigate possible complications of plerixafor in pediatric patients.

## Abbreviations

SDF1: Stromal cell-derived factor 1; CXCR4: CXC chemokine receptor 4; PBSC: Peripheral blood stem cell; G-CSF: Granulocyte colony stimulating factor.

## Conflict of interest

The authors declare that they have no competing interests.

## Authors' contributions

KTH collected and analyzed the patient data and wrote the manuscript, NHK, MSK collected the patient data, JWL, HK, KDP, HYS, HSA assisted the data interpretation, and HJK designed and coordinated the study. All authors have read and approved the final manuscript.
